# Periodontal manifestations of Langerhans cell histiocytosis: a systematic review

**DOI:** 10.1007/s00784-021-03873-0

**Published:** 2021-03-22

**Authors:** Julia C. Difloe-Geisert, Selina A. Bernauer, Noémie Schneeberger, Michael M. Bornstein, Clemens Walter

**Affiliations:** 1 Department of Periodontology, Endodontology and Cariology, University Center for Dental Medicine Basel (UZB), University of Basel, Mattenstrasse 40, 4058 Basel, Switzerland; 2 Department Oral Health & Medicine, University Center for Dental Medicine Basel (UZB), University of Basel, Basel, Switzerland

**Keywords:** Langerhans cell histiocytosis, Systemic diseases, Oral manifestations, Periodontal diseases

## Abstract

**Objectives:**

To explore the evidence of periodontal manifestations and treatment modalities in patients with Langerhans cell histiocytosis (LCH).

**Material and methods:**

A systematic literature search was performed and the criteria for PRISMA and risk of bias assessment were applied. Human clinical studies (≥10 patients) presenting patients with LCH and periodontal findings were considered for inclusion.

**Results:**

From 298 titles identified, six case series with a total of 1278 patients suffering from LCH were included. In these studies, oral symptoms were reported in a frequency ranging from 10 to 100%. Overall, in 216 patients (17%), oral symptoms were observed. Out of these patients, 49–100% demonstrated periodontal symptoms. The most common oral findings were pain, swelling, tooth loss/mobility, and bone lesions. Specific periodontal findings comprised varying frequencies of gingival ulcerations, increased pocket depths, and gingival bleeding. Treatment measures constituted of surgical curettage of bone lesions, soft tissue excision and/or tooth extractions, radiotherapy, systemic chemotherapy, or a combination of these approaches. Healing without recurrence of oral lesions was reported in most of the cases.

**Conclusions:**

The available evidence on periodontal manifestations in LCH patients is heterogeneous. Several oral and periodontal findings were reported and may occur as initial symptoms and/or at later stages of the disease.

**Clinical relevance:**

The dentist should be aware of possible oral involvement of systemic diseases such as LCH, and these manifestations may mimic periodontal disease.

**Supplementary Information:**

The online version contains supplementary material available at 10.1007/s00784-021-03873-0.

## Introduction

Langerhans cell histiocytosis (LCH; older term: histiocytosis X) is defined as a rare disease with an estimated annual incidence of 5 to 9 cases per million in children < 15 years of age [1–3], and 1 case per million in patients > 15 years of age [4]. The disease can occur at any age; however, it is most commonly diagnosed during childhood [1]. A slight predominance was reported in males [1, 2].

LCH is characterized by an excessive proliferation and accumulation of histiocytes (also called Langerhans cells) in various tissues [5–7]. This proliferation can lead to an infiltration of organs, thereby replacing the normal organ structure. Up to now, the understanding of LCH pathogenesis is still incomplete; however, neoplastic and inflammatory stimuli may contribute to disease progression. Histopathologically, infiltrates of histiocytes are present, with variable numbers of eosinophils, plasma cells, lymphocytes, and/or multinucleated giant cells [8]. The clinical presentation of LCH depends on the involved organs; e.g., bone, skin, lymph system, liver, lung, hematopoietic system, and/or oral tissues can be affected. Secondary complications may occur, such as diabetes insipidus following infiltration of the hypophysis. Historically, LCH was classified into the three clinical subtypes eosinophilic granuloma, Hand-Schüller-Christian disease, and Letterer-Siwe disease [5]. A current approach is to categorize LCH into a single-system form, i.e., a single organ is affected, and a multi-system form, i.e., several organs are involved [8]. Organ involvement may influence the progression and severity of disease, and a more favorable prognosis exists in patients with a single-system form [9].

In the current classification of periodontal and peri-implant diseases and conditions of 2018, LCH is listed as a “disorder that may be associated with progressive loss of the alveolar bone and increased mobility of affected teeth” [10, 11]. The strength of association was estimated as moderate. Therefore, the aim of the present systematic review was to assess the evidence of periodontal manifestations and treatment modalities in patients suffering from LCH.

## Material and methods

### Protocol

The present systematic review was accomplished adhering to PRISMA (Preferred Reporting Items for Systematic Reviews and Meta-Analyses) criteria (Online Resource 1) [12, 13].

### Outcome measures

The primary outcome measures were periodontal symptoms as assessed clinically or radiologically in patients suffering from LCH. Therapies applied and response of oral lesions to these therapeutic approaches were considered secondary outcome variables.

### Eligibility criteria

Publications were considered eligible for inclusion in this systematic review if the following parameters were presented:

1. Original in vivo study, i.e., randomized controlled clinical studies, controlled clinical studies, prospective or retrospective case series,
2. Study performed in humans,
3. Study including at least 10 patients [14],
4. Diagnosis of LCH as primary disease,
5. Presentation of healthy or diseased periodontal soft and hard tissues (i.e., gingiva and/or periodontal ligament and tooth-bearing parts of the alveolar bone), and
6. Publication in English, French, or German language.

### Information sources and search

The electronic databases MEDLINE and Embase via Ovid, Cochrane Central Register of Controlled Trials (CENTRAL), and the gray literature (http://www.opengrey.eu) were searched for studies published up to January 13, 2021. The search protocols within the different databases were applied and validated as identically as possible (Online Resource 2). The following search terms were applied: “histiocytosis” OR “Langerhans cell” OR “eosinophilic granuloma” OR “Hand Schuller” AND “periodont*” OR “oral disease” OR “oral involvement” OR “oral lesion” OR “oral manifestation” OR “dental disease” OR “dental involvement” OR “dental lesion” OR “dental manifestation.”

In addition, potentially relevant citations were harvested from the bibliographies of included studies and relevant reviews on the topic and examined for inclusion eligibility. The references resulting from the searches were entered in EndNote (Version X9, Clarivate Analytics, Philadelphia, PA, USA), and duplicates were removed.

### Study selection

**Fig. 1:**
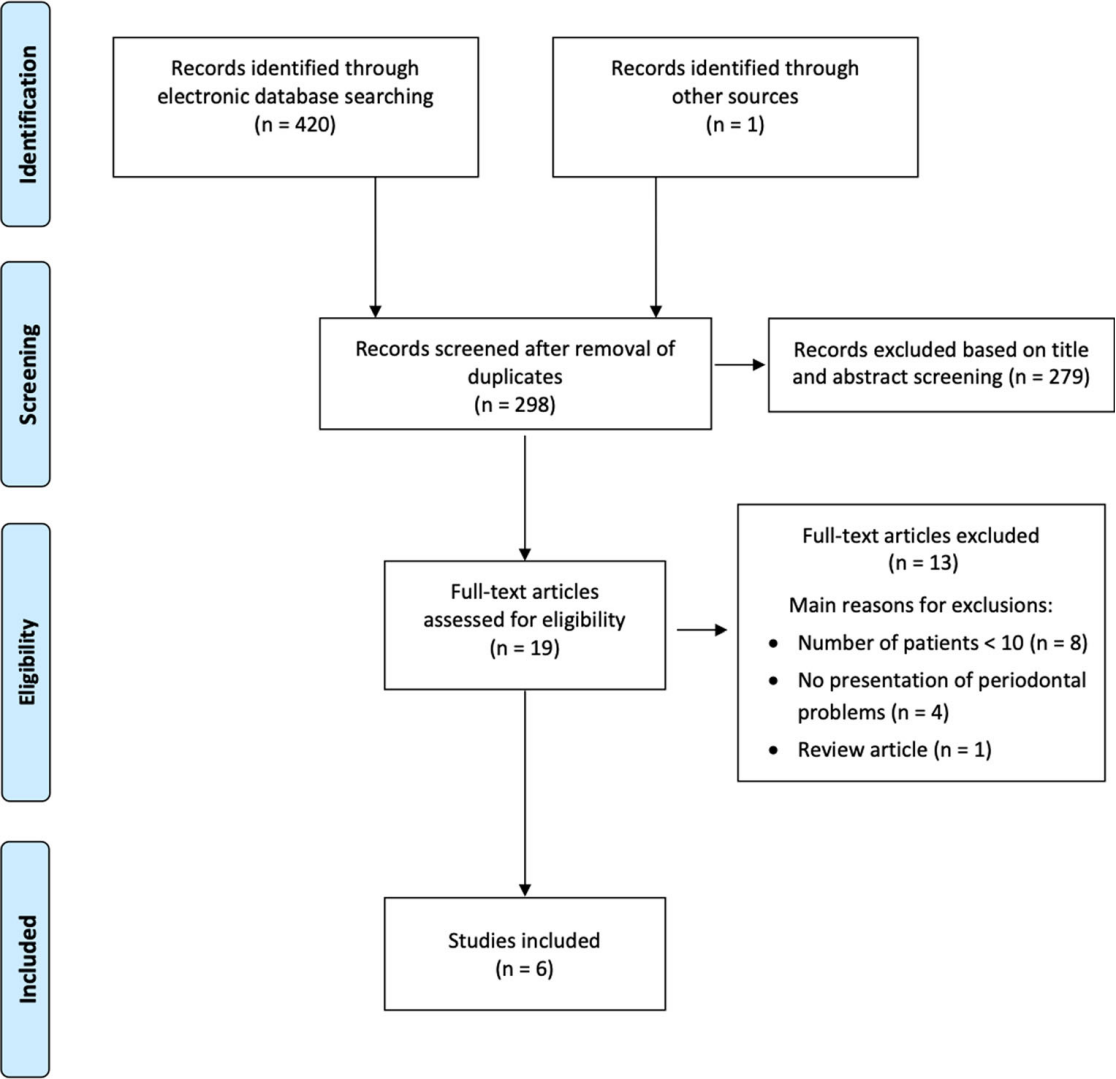
Selection process for the studies included [12]

Screening of records was performed independently by two of the authors (S. A. B. and J. C. D.). Cohen’s kappa score was calculated to assess inter-examiner agreement [15]. Eligibility assessment was performed through title and abstract analysis and secondly through full-text analysis (Fig. 1, Online Resource 3). From all studies of potential relevance, full-texts were obtained for independent assessment by the reviewers. Any disagreement was resolved by discussion among the two authors. The software system for recording decisions was Microsoft Excel (Version 16.44, Microsoft Corporation, Redmond, WA, USA).

### Data collection process

The following data of included studies were collected in duplicate by two independent reviewers using standardized data extraction files (Microsoft Word, Version 16.44, Microsoft Corporation, Redmond, WA, USA): characteristics of the study population, systemic manifestations, oral manifestations, treatment modalities, dropouts, duration, and results of follow-up oral examinations (Table 1).

**Table 1:** Characteristics and outcomes of the studies included

First author (year) country study design	*n* patients (*n* female) *n* (%) with oral symptoms	Mean age at diagnosis (range)	Systemic manifestations or involved organs		Oral manifestations				Therapy		Mean follow-up in years (range)	*n* dropouts *n* deaths	Oral follow-up	
				*n* patients (%*)	Unspecific	*n* patients (%*)	Periodontal	*n* patients (%*)		*n* patients (%**)			Result *n* patients (%**)	
Sedano (1969) [16] France, USA Case series	22 (9) 17 (77)	n.r. (3 mo to 45 yrs)	Anemia Bone lesions Diabetes Skin lesions	n.r. n.r. n.r. n.r.	Bone lesions Foetor ex ore Tooth loss Tooth mobility	n.r. n.r. n.r. n.r.	Alveolar bone loss Gingival bleeding Gingival necrosis Gingival swelling Gingival ulceration	n.r. n.r. n.r. n.r. n.r.	RT SCT	n.r. n.r.	n.r.	n.r. 4	n.r.	
Sigala (1972) [17] USA Case series	50 (26) 18 (36)	n.r. (1 mo to 64 yrs)	Bone lesions Diabetes insipidus Exophthalmos Hematopoietic system Hepatosplenomegaly Lymphadenopathy Otitis media Skin lesions	38 (76) 14 (28) 11 (22) 11 (22) 23 (46) 18 (36) 19 (38) 21 (42)	Bone lesions Pain Tooth mobility/loss	18 (36) 8 (16) 18 (36)	Gingival ulceration	18 (36)	n.r.	n.r.		n.r. n.r.	n.r.	
Hartman (1980) [18] USA Case series	1120^e^ (n.r.) 114 (10)	n.r. (2 w to 53 yrs)	Anemia^n^ Bone^o^ Diabetes insipidus^n^ Exophthalmos^n^ Fever^n^ Hepatosplenomegaly Leukocytosis^n^ Lung^o^ Lymphadenopathy^n^ Mental retardation^n^ Otitis media^n^ Salivary glands^o^ Skin lesions^n^	17 (21) 114^a^ (n.a.) 18 (22) 11 (13) 8 (10) 10 (12) 2 (2) 14 (13) 18 (22) 2 (2) 9 (11) 3 (3) 14 (17)	Biting problems^m^ Bone lesions^k^ Bone lesions^k^ Foetor ex ore^m^ Mouth floor lesions^k^ Mucosa lesions^d,k^ Oral ulceration^m^ Pain^m^ Paresthesia^m^ Poor healing^m^ Sinus lesions^k^ Swelling^m^ Tooth mobility^m^	9 (9) 164^b^ (n.a.) 52^c^ (n.a.) 12 (12) 6 (5) 4 (4) 19 (19) 46 (46) 4 (4) 13 (13) 5 (4) 59 (59) 41 (41)	Gingival bleeding^m^ Gingival lesions^k^ Gingivitis^m^ NUG^m^	6 (6) 79 (69) 43 (43) 7 (7)	RT RT + SC or STE SC or STE SCT	20 (23) 21 (24) 42 (48) 5 (6)	8.3 (0.5-29)	26 19^f^	Healing Relapse Residual	72 (82) 14 (16) 2 (2)
Mínguez (2004) [19] Spain Case series	10 (4) 10 (100)	1.7 (4 mo to 3 yrs)	Adenopathy Anemia Bone Diabetes insidipus Exophthalmos Fever Hepatomegaly Lung Otitis Skin lesions Weight loss	4 (40) 3 (30) 17^g^ (n.a.) 5 (50) 3 (30) 6 (60) 2 (20) 3 (30) 7 (70) 9 (90) 3 (30)	Aphthae Bone lesions Candidiasis Foetor ex ore Pain Swelling Tooth loss Tooth mobility	9 (90) 6 (60) 4 (40) 1 (10) 2 (20) 3 (30) 5 (50) 2 (20)	Gingival bleeding Ulceration/ Bleeding of gums, mouth floor and jugal mucosa	7 (70) 1 (10)	SCT SCT + SC SCT + SC + C SCT + SC + RT	6 (60) 1 (10) 1 (10) 2 (20)	6 (1-11)	1 1^f^	Healing Relapse	7 (78) 2 (22)
Annibali (2009) [20] Italy Case series	31 (16) 12^p^ (39)	37.3 (18 to 73 yrs)	Bone^h^ Hypophysis Lung Lymph system Skin Vulva	18 (58) 5 (16) 15 (48) 3 (10) 2 (6) 2 (6)	Bone lesions Pain Poor healing Tooth mobility	12 (39) 10 (32) 1 (3) 2 (6)	Gingival bleeding Gingival and/or alveolar mucosa ulceration Increased PPD, CAL, and/or bone loss	8 (26) 6 (19) 6 (19)	SC + TE SC or STE SC + TE + IFN SC + TE + STE SCT	5 (42) 4 (33) 1 (8) 1 (8) 1 (8)	5.7 (0.6-18)	6^f^ 2^f^	Healing Relapse	10 (83) 2 (17)
Capodiferro (2020) [21] Italy Case series	45 (19) 45^q^ (100)	4.8 (0 to 18 yrs)	Cutaneous rush Bone lesions^r^ Diabetes insidipus Exophtalmos Lymphadenopathy^s^ Otitis media or externa	9 (20) 14 (31) 8 (18) 2 (4) 12 (27) 4 (9)	Bone lesions Mandible fracture Paresthesia Premature tooth loss with dislocation of permanent teeth follicles Palatal mucosa lesion	9 (20) 6 (13) 3 (7) 9 (20) 6 (13)	Gingival lesions (inflamed, hyperplastic and/or ulcerated) Gingival swelling in patients with single jawbone lesions Tooth mobility and/or dislocation with gingival probing and periodontal bone loss	22 (49) 8 (18) 18 (40)	SC SCT C RT	n.r. n.r. (80^#^) n.r. (> 50^#^) 3 (7)	> 15 yrs in 20 patients	n.r. 4	Healing Relapse	n.r. 8 (18)

*C*, intralesional corticoids; *CAL*, clinical attachment loss; *IFN*, α-interferon; *n*, number; *n.a.*, not applicable; *n.r*., not reported; *mo*, months; *NUG*, necrotizing ulcerative gingivitis; *PPD*, probing pocket depth; *RT*, radiotherapy; *SC*, surgical curettage; *SCT*, systemic chemotherapy; *STE*, soft tissue excision; *TE*, tooth extraction; *w*, weeks; *yrs*, years; ^a^, sum of bone lesions: skull lesions (*n*=42), lower extremity (*n*=31), upper extremity (*n*=15), ribs (*n*=15), and spine (*n*=11); ^b^, sum of mandibular bone lesions: posterior (*n*=103), anterior (*n*=49), and not stated (*n*=12); ^c^, sum of maxillary bone lesions: posterior (*n*=31), anterior (*n*=18), and not stated (*n*=3); ^d^, buccal mucosa; ^e^, 1120 patients screened for oral involvement, and data of 114 patients (20 females) with oral symptoms presented; ^f^, included in results of follow-up; ^g^, sum of bone lesions: long bone (*n*=7), cranial (*n*=6) and mastoid bone (*n*=4); ^h^, including skull (*n*=11), clavicle (*n*=1), scapula (*n*=2), hip (*n*=1), ilium (*n*=1), ribs (*n*=2), tibis (*n*=2), omerus (*n*=1), and femur (*n*=1); ^k^, investigated in 114 patients; ^m^, investigated in 100 patients; ^n^, investigated in 82 patients; ^o^, investigated in 112 patients; ^p^, onset during follow-up of LCH treatment; ^q^, exclusively patients included with disease onset in the head and neck region; ^r^, skull lesions; ^s^, submental, submandibular and/or lateral-cervical lymph nodes *, calculated by the authors, percentages refer to total n patients within each study; **, calculated by the authors, percentages refer to n patients receiving treatment within each study; ^#^, reported in the publication without absolute number

### Frequency distribution of oral LCH manifestations

A frequency distribution of each finding (e.g. involved organ systems and symptoms) was calculated and shown as an absolute number and percentage [22]. Findings not reported in any patient of a case series were interpreted as not investigated and excluded from the respective frequency calculations. Thus, the reference number of patients differed from finding to finding. The maximum possible reference number of patients was the sum of all included patients (if a finding was examined in all included case series).

### Quality assessment of included studies

The risk of bias of included studies was evaluated by the checklist from Moga et al. [23], including 18 items of the following topics: (a) study objective; (b) study population; (c) intervention and co-intervention; (d) outcome measure; (e) statistical analysis; and (f) results and conclusion (Online Resource 4). Considering the adequacy in the respective case series, the items were graded and the percentage of positively graded items was calculated [14, 24] (Online Resource 5).

## Results

### Study selection

A total of 298 studies were identified by electronic and hand search (Fig. 1). After title and abstract screening (inter-examiner Cohen’s kappa score = 0.75), the full texts of 19 studies were screened for possible inclusion (Online Resource 3). Finally, six articles published between 1969 and 2020 fulfilled the inclusion criteria and thus remained for analysis [16–21]. All included studies were case series without controls.

### Summary of study characteristics

#### Population

The studies included a total of 1278 patients (Table 1). In one study, 1120 records of LCH patients were screened for patients with oral symptoms and subsequently, the data of 114 patients presenting oral involvement were included and presented [18]. Thus, detailed data were available from six studies with a total of 272 patients.

All studies provided data about the proportion of males and females; i.e., out of 272 patients, 94 (35 %) were females and 178 (65 %) males. The age of the patients ranged between 2 weeks and 73 years at the time of LCH diagnosis. Three studies provided the mean age of patients that amounted to 1.7, 4.8, and 37.3 years, respectively [19–21].

A total of 131 patients (48 %) suffered from a single-system disease pattern or eosinophilic granuloma, and 137 patients (50 %) were diagnosed with a multi-system disease pattern or disseminated histiocytosis, Hand-Schüller-Christian, or Letterer-Siwe disease. In four patients (2 %), disease pattern was not specified [16].

In all studies, systemic manifestations were described (Table 1). Involvement of the skeletal system was documented as extraoral bone lesions in 70 out of 126 patients (56 %) [17, 20, 21]. In addition, two studies differentiated between various bone types involved [18, 19]. Subsequent to bone lesions, the involvement of the following three organs and/or symptoms was most frequently reported: skin or skin lesions (55 out of 218 patients, 25 %), hypophysis or diabetes insipidus (50 out of 218 patients, 23 %), and lymph system or lymphadenopathy (43 out of 173 patients, 25 %). Additional organs affected and/or related symptoms are shown in Table 1.

#### Primary outcome: periodonal manifestations

A total of 216 out of 1278 patients (17 %) demonstrated oral involvement (Table 1). Periodontal diagnoses were gingivitis (43 out of 100 patients, 43 %) and necrotizing ulcerative gingivitis (7 out of 100 patients, 7 %). In addition, periodontal findings comprised gingival bleeding (21 out of 141 patients, 15 %), gingival ulcerations or lesions (126 out of 199 patients, 63 %), gingival swelling (8 out of 45 patients, 18%), and increased pocket depths and/or clinical attachment loss (24 out of 76 patients, 32 %).

The most commonly reported oral symptoms, not unambiguously related to periodontal inflammation, included tooth loss and/or tooth mobility (76 out of 236 patients, 32 %), oral pain (66 out of 191 patients, 35 %), and swelling (62 out of 110 patients, 56 %). Alveolar bone lesions were found in 45 out of 136 patients (33 %). In addition, one study differentiated between the regions of bone lesions and observed most lesions in posterior regions of the mandible (103 out of 114 patients, 90%) [18]. Further oral symptoms are presented in Table 1.

#### Secondary outcome: treatment modalities and response to treatment

Three of the included studies reported the number of patients receiving systemic and/or local treatment measures [18–20]. In one of these studies, patients were already receiving a systemic chemotherapy when oral symptoms were detected [20]. These patients received an additional second-line therapy. Treatment measures included systemic chemotherapy (12 out of 110 patients, 11 %) with vinblastine, corticoids, and/or methotrexate; radiotherapy (20 out of 110 patients, 18 %); oral surgery (52 out of 110 patients, 47 %) including curettage, soft tissue excision, and/or tooth extractions; or a combination of these therapeutic approaches (26 out of 110 patients, 24 %).

The mean duration of follow-up was given in three out of six studies and varied from 6 months to 29 years [18–20]. The mean observation period amounted to 5.7, 6, and 8.3 years, respectively. Dropouts were reported in three out of six studies [18–20]. The main reason for dropouts was a too-long distance between residence and study center and thus decision for a nearer clinic for follow-up in six patients [20]. In 27 patients, the reasons for dropouts were not provided [18, 19]. Additionally, 30 patients died during follow-up [16, 18–21]. In 16 patients, death was related to LCH, while 14 patients died due to other or unknown reasons.

In 89 out of 109 patients (82 %), healing after therapeutic interventions of oral lesions was documented during follow-up [18–20]. While 26 out of 154 patients (17 %) developed new oral manifestations, two patients (2 %) showed residual oral lesions.

### Quality assessment

The assessment of the risk of bias of the included case series is illustrated in the Online Resource 5 and was based on the checklist from Moga et al. [23] to evaluate the methodological and reporting quality of case series. Percentage of positively graded items relevant for quality assessment ranged from 24 to 65 %; i.e., high to moderate risk of bias was present in all included case series.

## Discussion

The purpose of the present systematic review was to assess periodontal manifestations and treatment modalities in LCH patients. Six case series with a total of 1278 patients suffering from LCH were included, and absolute and relative frequencies of symptoms calculated. The frequency of oral symptoms was heterogenous and ranged from 10 to 100 %. In 216 patients, oral symptoms were reported. Oral lesions affected both hard and soft tissues. Periodontal findings comprised most frequently gingival diseases, i.e., gingival ulcerations or lesions and gingival bleeding. Increased pocket depths and attachment loss were reported in two case series. The remaining oral findings were mostly unspecific with a possible, but not mandatory, relation to periodontal diseases. Among these were with varying frequencies of oral pain, oral swelling, tooth loss or tooth mobility, foetor ex ore, and biting problems. Bone lesions occurred most frequently and affected predominantly the posterior regions of the mandible. From a radiographic point of view, a similarity of bone lesions to a wide spectrum of diseases was described, such as odontogenic cysts, certain benign and malignant tumors, periapical lesions, and periodontal bone loss.

In the current classification of periodontal and peri-implant diseases and conditions, LCH was recently listed as group of “conditions which affect the periodontal apparatus independently of dental plaque/biofilm-induced inflammation” [10, 11]. The authors reported that LCH may lead to periodontal inflammation and severe periodontal destruction with increased pocket depths, alveolar bone loss, and tooth loss. The strength of association was estimated as moderate.

Herein, we aimed to systematically evaluate the available evidence and identified six case series with pronounced heterogeneity regarding patient and disease characteristics and methodology. In this context, the following aspects may be discussed:

1. The age of patients in the included case series showed a wide range between 2 weeks and 73 years. In one study, 40% of patients were up to 9 years old [18], and in three studies, the majority of patients constituted children up to 6 years [16, 17, 19]. In contrast, Annibali et al. [20] included only patients ≥ 18 years. These patients were already receiving active treatment for systemic disease control when oral examinations were performed.
2. Patients showed different disease stages and duration. In 64 patients, oral lesions were reported to present the first manifestation of LCH [16, 17, 19, 21]. In two studies, 22 out of 41 patients developed oral lesions after initial LCH diagnosis and under systemic treatment [19, 20]. The length of follow-up varied between 6 months and 29 years in the included case series. LCH may clinically manifest itself in several acute phases and oral symptoms may newly develop or return in the course of disease. Thus, oral lesions may constitute a first sign of initial disease manifestation or a sign of disease reactivation.
3. LCH is typically characterized by a very heterogeneous clinical picture [8]. In the studies included, patients also presented different disease severities and patterns, as revealed by a wide range of systemic comorbidities and complications. While 131 (48 %) patients suffered from a single-system disease, 137 (50 %) were diagnosed with a multi-system disease pattern. Sedano et al. [16] found oral lesions as the only sign of disease in three patients. In contrast, all patients assessed by Minguez et al. [19] demonstrated a chronic disseminated form of LCH. The remaining studies included patients with varying disease patterns. In 16 patients, disease showed even a fatal course, and patients died following severe sequelae related to their underlying disease [16, 18–21]. Two studies differentiated between different LCH disease patterns when reporting on oral involvement [18, 20]. One of these observed that unifocal oral involvement (one lesion) was associated with a multi-systemic disease pattern while multifocal oral lesions (> 1 lesion) were related to unisystemic disease [20]. However, there was no explanation for this association.
4. The case series included showed a moderate to high risk of reporting bias (Online Resource 5). Outcome parameters were mainly not predefined and reported for the first time in the results section of the studies. Thus, it seems difficult for the reader to distinguish between findings not investigated and findings investigated, but not detected and therefore not reported. To address this, we considered findings not reported in any patient as not investigated. The reporting frequency of different oral symptoms varied. While gingival ulceration was evaluated in all included case series (272 patients), probing pocket depths and clinical attachment loss were examined in two studies (76 patients) [20, 21]. Consequently, the reference number of patients was adapted for each symptom when absolute or relative frequencies were calculated. A further source of bias may be the different sizes of included case series, ranging from 10 patients [19] to 1120 patients [18]. Out of the 1120 patients, however, only the data of patients with oral symptoms were presented in detail, i.e., of 114 patients.
5. In addition, severity and/or quantification of outcome parameters were not reported. For example, tooth loss was described in four studies [16, 17, 19, 21]. However, no data on the number of missing teeth were available. Similarly, Annibali et al. [20] recorded probing pocket depths and clinical attachment loss in 6-month intervals and summarized findings as periodontal damage found in six out of 31 patients.
6. Finally, no details were provided on the clinical and/or radiological examination methods used to assess outcome parameters. In one study, patients were assessed by different clinicians at follow-up examinations [20]. Information about examiner calibration was lacking. Thus, the reproducibility and reliability of applied methods appear difficult to estimate.

Depending on the type and severity of oral lesions, patient quality of life may be significantly impaired [25]. Four out of six studies reported on oral pain. Sigala et al. [17] described pain as the chief complaint in eight out of 18 patients with oral symptoms. Furthermore, 42% of patients complained of negative outcomes on quality of life due to oral lesions [20].

The reported treatment for oral lesions was surgical curettage of bone lesions, associated with tooth extractions and/or excision of involved soft tissues. The prognosis was favorable and most lesions disappeared, as evidenced by the overall remission rate of 82%. Non-surgical periodontal treatment was not reported in any of the included case series. As LCH was described to affect the periodontal tissues independently of dental plaque/biofilm-induced inflammation [10, 11], mechanical biofilm removal, and/or scaling and root planning of diseased periodontia may be not expected as first-line therapy of oral lesions. Nevertheless, in case of periodontal inflammation, we suggest that oral treatment may be supported by measures of periodontal infection control, e.g., oral hygiene instruction and biofilm management and complemented by regular periodontal controls.

## Conclusions

Based on the evidence from the case series with at least 10 patients, oral symptoms including periodontal manifestations typically seen for periodontal diseases may occur as initial and/or long-term symptoms and with varying frequencies (10–100 %) in patients with LCH. Thereby, LCH may mimic more common pathologies such as periodontal diseases. A thorough clinical inspection of the oral cavity for affected patients including the periodontal tissues is mandatory to detect any oral manifestation at an early stage. In case of oral and periodontal symptoms not or hardly explainable by other factors (e.g., insufficient oral hygiene) and/or a lack of response to conventional treatments, dentists should generally consider the possibility of an underlying systemic disease and initiate further diagnostic procedures by referral to a specialist.

## Supplementary information

ESM 1 (DOC 64 kb)

ESM 2 (DOCX 16 kb)

ESM 3 (DOCX 21 kb)

ESM 4 (DOCX 17 kb)

ESM 5 (DOCX 23 kb)
