# Supplementary material for: Periodontal manifestations of Langerhans cell histiocytosis: a systematic review
Source: Clin Oral Investig. 2021 Mar 22;25(6):3341–9. doi: 10.1007/s00784-021-03873-0 (PMC8137606; doi:10.1007/s00784-021-03873-0)
Supplement: Supplementary file 2 — (DOCX 16 kb) [file 784_2021_3873_MOESM2_ESM.docx]

**Periodontal manifestations of Langerhans cell histiocytosis: a systematic review**

Clinical Oral Investigations

Julia C. Difloe-Geisert^1^*, Selina A. Bernauer^1^*, Noémie Schneeberger^1^, Michael M. Bornstein^2^, Clemens Walter^1‡^

^1^Department of Periodontology, Endodontology and Cariology, University Center for Dental Medicine (UZB), University of Basel, Switzerland

^2^Department Oral Health & Medicine, University Center for Dental Medicine (UZB), University of Basel, Switzerland

* Julia C. Difloe-Geisert, Selina A. Bernauer: Shared first authorship.

^‡^**Corresponding author:**

Prof. Dr. med. dent. Clemens Walter

Department of Periodontology, Endodontology and Cariology

University Center for Dental Medicine (UZB), University of Basel

Mattenstrasse 40

4058 Basel (Switzerland)

Phone: +41 61 2672628

Email: [clemens.walter@unibas.ch](mailto:clemens.walter@unibas.ch)

**Online Resource 2** Electronic search strategy for the databases MEDLINE and Embase (via Ovid).

| - **No.** | - **Search syntax** |
| --- | --- |
|  |  |
| - #1 | - (histiocytosis or Langerhans-cell or eosinophilic granuloma or Hand-Schuller).mp. [mp= ti, ab, hw, tn, ot, dm, mf, dv, kw, fx, dq, nm, kf, ox, px, rx, an, ui, sy] |
| - #2 | - periodont*.mp. [mp=ti, ab, hw, tn, ot, dm, mf, dv, kw, fx, dq, nm, kf, ox, px, rx, an, ui, sy] |
| - #3 | - ((oral or dental) adj1 (disease or involvement or lesion or manifestation)).mp.[mp=ti, ab, hw, tn, ot, dm, mf, dv, kw, fx, dq, nm, kf, ox, px, rx, an, ui, sy] |
| - #4 | - 2 or 3 |
| - #5 | 1 and 4 |
