# Supplementary material for: Periodontal manifestations of Langerhans cell histiocytosis: a systematic review
Source: Clin Oral Investig. 2021 Mar 22;25(6):3341–9. doi: 10.1007/s00784-021-03873-0 (PMC8137606; doi:10.1007/s00784-021-03873-0)
Supplement: Supplementary file 3 — (DOCX 21 kb) [file 784_2021_3873_MOESM3_ESM.docx]

**Periodontal manifestations of Langerhans cell histiocytosis: a systematic review**

Clinical Oral Investigations

Julia C. Difloe-Geisert^1^*, Selina A. Bernauer^1^*, Noémie Schneeberger^1^, Michael M. Bornstein^2^, Clemens Walter^1‡^

^1^Department of Periodontology, Endodontology and Cariology, University Center for Dental Medicine (UZB), University of Basel, Switzerland

^2^Department Oral Health & Medicine, University Center for Dental Medicine (UZB), University of Basel, Switzerland

* Julia C. Difloe-Geisert, Selina A. Bernauer: Shared first authorship.

^‡^**Corresponding author:**

Prof. Dr. med. dent. Clemens Walter

Department of Periodontology, Endodontology and Cariology

University Center for Dental Medicine (UZB), University of Basel

Mattenstrasse 40

4058 Basel (Switzerland)

Phone: +41 61 2672628

Email: [clemens.walter@unibas.ch](mailto:clemens.walter@unibas.ch)

**Online Resource 3** Studies excluded based on full-text analysis, and reasons for exclusion.

| First author (year of publication) | Reason for exclusion |
| --- | --- |
| Fasulo et al. (1966) | 1 |
| Moskow et al. (1971) | 1 |
| Carraro et al. (1972) | 1 |
| Winther et al. (1972) | 1 |
| Hausamen et al. (1973) | 1 |
| Moorthy et al. (1986) | 1 |
| Moghadam et al. (1991) | 1 |
| Kusumakumary et al. (2000) | 2 |
| Bartnick et al. (2002) | 2 |
| Haupt et al. (2004) | 2 |
| Moghadam et al. (2015) | 2 |
| Khonsari & Ruhin (2016) | 1 |
| Hanisch et al. (2019) | 3 |
| 1, number of patients <10; 2, no examination of periodontal tissues; 3, review article. | |

**References**

1. Fasulo CP, van Gaasbeek JB. Hand-Schüller-Christian disease. Medical and oral surgical problems involved. Oral Surg Oral Med Oral Pathol. 1966;22;555-63.
2. Moskow R, Levine LJ, Marin A. Multifocal eosinophilic granuloma simulating periodontal disease. NY State Dent J. 1971;37:607-11.
3. Carraro JJ, Sznajder N, Barros R, Lalis RM. Periodontal involvement in eosinophilic granuloma. J Periodontol. 1972;43:427-32.
4. Winther JE, Fejerskov O, Philipsen HP. Oral manifestations of histiocytosis X. Acta Derm Venereol. 1972;52:75-9.
5. Hausamen JE, Fesseler A. [Differential diagnosis of eosinophilic granuloma and deep marginal periodontitis.] Dtsch Zahnarztl Z. 1973;28:270-5.
6. Moorthy AP. Eosinophilic granuloma manifesting as a periodontal problem. Br Dent J. 1986;161:66-7.
7. Moghadam BK, Saedi S, Gier RE. Adult-onset multifocal histiocytosis X presenting as a periodontal problem. J Oral Maxillofac Surg. 1991;49:417-9.
8. Kusumakumary P, James FV. Permanent disabilities in childhood survivors of Langerhans cell histiocytosis. Pediatr Hematol Oncol. 2000;17:375-81.
9. Bartnick A, Friedrich RE, Roeser K, Schmelzle R. Oral Langerhans cell histiocytosis. J Craniomaxillofac Surg. 2002;30:91-6.
10. Haupt R, Nanduri V, Calevo MG, et al. Permanent consequences in Langerhans cell histiocytosis patients: a pilot study from the histiocyte society-late effects study group. Pediatr Blood Cancer. 2004;42:438-44.
11. Atarbashi Moghadam S, Lotfi A, Piroozhashemi B, Mokhtari S. A retrospective analysis of oral Langerhans cell histiocytosis in an Iranian population: a 20-year evaluation. J Dent (Shiraz). 2015;16:274-7.
12. Khonsari RH, Ruhin B. Images in clinical medicine. Loose teeth and excessive thirst. N Engl J Med. 2016;374:e25.
13. Hanisch M, Hoffmann T, Bohner L, et al. Rare diseases with periodontal manifestations. Int J Environ Res Public Health. 2019;16:e867.
