# Supplementary material for: Periodontal manifestations of Langerhans cell histiocytosis: a systematic review
Source: Clin Oral Investig. 2021 Mar 22;25(6):3341–9. doi: 10.1007/s00784-021-03873-0 (PMC8137606; doi:10.1007/s00784-021-03873-0)
Supplement: Supplementary file 4 — (DOCX 17 kb) [file 784_2021_3873_MOESM4_ESM.docx]

**Periodontal manifestations of Langerhans cell histiocytosis: a systematic review**

Clinical Oral Investigations

Julia C. Difloe-Geisert^1^*, Selina A. Bernauer^1^*, Noémie Schneeberger^1^, Michael M. Bornstein^2^, Clemens Walter^1‡^

^1^Department of Periodontology, Endodontology and Cariology, University Center for Dental Medicine (UZB), University of Basel, Switzerland

^2^Department Oral Health & Medicine, University Center for Dental Medicine (UZB), University of Basel, Switzerland

* Julia C. Difloe-Geisert, Selina A. Bernauer: Shared first authorship.

^‡^**Corresponding author:**

Prof. Dr. med. dent. Clemens Walter

Department of Periodontology, Endodontology and Cariology

University Center for Dental Medicine (UZB), University of Basel

Mattenstrasse 40

4058 Basel (Switzerland)

Phone: +41 61 2672628

Email: [clemens.walter@unibas.ch](mailto:clemens.walter@unibas.ch)

**Online Resource 4** Criteria for assessing the methodological and reporting quality of case series studies according to Moga et al. [17].

| **Major Components** | **Judgment** |
| --- | --- |
|  |  |
| 1. Is the hypothesis/aim/objective of the study clearly stated? | Yes, Unclear, No |
| 1. Are the characteristics of the participants included in the study described? | Yes, Partially reported, No |
| 1. Were the cases collected in more than one centre? | Yes, Unclear, No |
| 1. Are the eligibility criteria (i.e. inclusion and exclusion criteria) for entry into the study clearly stated? | Yes, Partially reported, No |
| 1. Were participants recruited consecutively? | Yes, Unclear, No |
| 1. Did participants enter the study at a similar point in the disease? | Yes, Unclear, No |
| 1. Was the intervention of interest clearly described? | Yes, Partially reported, No |
| 1. Were additional interventions (co-interventions) reported in the study? | Yes, Unclear, No |
| 1. Are the outcome measures established a priori? | Yes, Partially reported, No |
| 1. Were the relevant outcomes measured with appropriate objective and/or subjective methods? | Yes, Unclear, No |
| 1. Were the relevant outcomes measured before and after the intervention? | Yes, Unclear, No |
| 1. Were the statistical tests used to assess the relevant outcomes appropriate? | Yes, Unclear, No |
| 1. Was the length of follow-up reported? | Yes, Unclear, No |
| 1. Was the loss to follow-up reported? | Yes, Unclear, No |
| 1. Does the study provide estimates of the random variability in the data analysis of relevant outcomes? | Yes, Unclear or partially reported, No |
| 1. Are the adverse events related with the intervention reported? | Yes, Partially reported, No |
| 1. Are the conclusions of the study supported by results? | Yes, Partially reported, No |
| 1. Are both competing interests and sources of support for the study reported? | Yes, Partially reported, No |
