# Supplementary material for: Periodontal manifestations of Langerhans cell histiocytosis: a systematic review
Source: Clin Oral Investig. 2021 Mar 22;25(6):3341–9. doi: 10.1007/s00784-021-03873-0 (PMC8137606; doi:10.1007/s00784-021-03873-0)
Supplement: Supplementary file 5 — (DOCX 23 kb) [file 784_2021_3873_MOESM5_ESM.docx]

**Periodontal manifestations of Langerhans cell histiocytosis: a systematic review**

Clinical Oral Investigations

Julia C. Difloe-Geisert^1^*, Selina A. Bernauer^1^*, Noémie Schneeberger^1^, Michael M. Bornstein^2^, Clemens Walter^1‡^

^1^Department of Periodontology, Endodontology and Cariology, University Center for Dental Medicine Basel (UZB), University of Basel, Switzerland

^2^Department Oral Health & Medicine, University Center for Dental Medicine Basel (UZB), University of Basel, Switzerland

* Julia C. Difloe-Geisert, Selina A. Bernauer: Shared first authorship.

^‡^**Corresponding author:**

Prof. Dr. med. dent. Clemens Walter

Department of Periodontology, Endodontology and Cariology

University Center for Dental Medicine Basel (UZB), University of Basel

Mattenstrasse 40

4058 Basel (Switzerland)

Phone: +41 61 2672628

Email: [clemens.walter@unibas.ch](mailto:clemens.walter@unibas.ch)

**Online Resource 5** Quality assessment of included studies according to Moga et al. [17].

| *Major Components* | Sedano  et al.  (1969)^19^ | Sigala  et al.  (1972)^23^ | Hartman  (1980)^20^ | Mínguez  et al.  (2004)^21^ | Annibali  et al.  (2009)^22^ | Capodiferro  et al.  (2020)^25^ |
| --- | --- | --- | --- | --- | --- | --- |
| 1. Is the hypothesis/aim/objective of the study clearly stated? | + | + | + | + | + | + |
| 1. Are the characteristics of the participants included in the study described? | + | + | + | + | + | + |
| 1. Were the cases collected in more than one centre? | + | - | - | - | - | + |
| 1. Are the eligibility criteria (i.e. inclusion and exclusion criteria) for entry into the study clearly stated? | - | - | - | - | - | + |
| 1. Were participants recruited consecutively? | U | + | U | U | + | + |
| 1. Did participants enter the study at a similar point in the disease? | U | U | U | U | U | + |
| 1. Was the intervention of interest clearly described? | - | - | + | + | + | + |
| 1. Were additional interventions (co-interventions) reported in the study? | - | - | + | + | + | + |
| 1. Are the outcome measures established a priori? | - | - | - | - | + | - |
| 1. Were the relevant outcomes measured with appropriate objective and/or subjective methods? | U | U | U | U | U | U |
| 1. Were the relevant outcomes measured before and after the intervention? | - | - | + | + | + | U |
| 1. Were statistical tests used to assess the relevant outcomes appropriate? | n.a. | n.a. | n.a. | n.a. | n.a. | n.a. |
| 1. Was the length of follow-up reported? | - | - | + | + | + | - |
| 1. Was the loss to follow-up reported? | - | - | + | + | + | - |
| 1. Does the study provide estimates of the random variability in the data analysis of relevant outcomes? | - | - | - | - | - | - |
| 1. Are the adverse events related with the intervention reported? | - | - | - | - | - | - |
| 1. Are the conclusions of the study supported by results? | + | + | + | + | + | + |
| 1. Are both competing interests and sources of support for the study reported? | + | - | - | - | + | + |
| Percentage of positively graded items | 29 % | 24 % | 47 % | 47 % | 65 % | 59 % |
| +, yes; -, no; n.a., not applicable; P, partially reported; U, unclear. | | | | | |  |
